# Gender Differences in Depression in the General Population of Indonesia: Confounding Effects

**DOI:** 10.1155/2021/3162445

**Published:** 2021-06-24

**Authors:** Andi Agus Mumang, Saidah Syamsuddin, Ida Leida Maria, Irawan Yusuf

**Affiliations:** ^1^Medical Faculty Postgraduate (Doctoral) Program, Hasanuddin University, Makassar, South Sulawesi, Indonesia; ^2^Department of Psychiatry, Medical Faculty, Hasanuddin University, Makassar, South Sulawesi, Indonesia; ^3^Department of Epidemiology, Public Health Faculty, Hasanuddin University, Makassar, South Sulawesi, Indonesia; ^4^Department of Physiology, Medical Faculty, Hasanuddin University, Makassar, South Sulawesi, Indonesia

## Abstract

**Background:**

Research findings on gender differences in depression are inconsistent. This study investigated gender and depression in the Indonesian population and considered possible confounding effects.

**Methods:**

This was a cross-sectional study. Participants completed the following self-report measures: demographic characteristic questions, the Cultural Orientation Scale, and the Center for Epidemiological Studies Depression Scale. Gender differences in depression were examined using a generalized linear model.

**Results:**

After withdrawals, 265 men and 243 women remained. Women and men did not differ in overall scores and four-factor depression symptoms even after adjusting for cultural orientation and demographic confounding factors, except for the depression symptoms “crying,” “cannot get going,” and “people were unfriendly.” Gender differences in depression became significant after adjusting for stereotypical symptom variance. Men reported being lonelier than women.

**Conclusions:**

Possible confounding effects on the association between gender and depression are methodological issues, cultural orientation transition, and stereotypical symptoms. Low depression scores found for gender may reflect *dimension-counterpart coping strategies*.

## 1. Introduction

Findings on gender differences in depression are inconsistent. Some studies indicate that women have a higher prevalence of depression than men [1–5], whereas others report no gender differences in depression [6–8]. There is also evidence that more men than women report depression in some situations [9, 10].

There are several explanations offered for gender differences in depression, particularly for why women report more depression [4, 11–13]. However, these explanations may not take into account the above-mentioned conflicting findings (see also reviews of Parker and Brotchie [14] and Piccinelli and Wilkinson [15]), which indicate inconsistencies in gender differences in depression.

Gender differences may arise from constraints and flexibility in gender socialization, which may correlate with the psychodynamics of depression [9, 16]. Socialization constraints are shaped by gender-typed attributes (e.g., prototype or stereotype); flexibility refers to contextual influences (e.g., sociocultural aspects) [16]. These factors have implications for confounding effects on gender differences in depression. Adherence to gender-congruent stereotypes [17, 18] may lead to differences in prejudice [19] and cultural orientation (i.e., arising from the interaction between horizontal–vertical and individualism–collectivism dimensions) [20, 21]. The horizontal–vertical dimension reflects equality and hierarchy [22]. Therefore, gender differences in depression may reflect gender socialization, which may shape responsiveness to sociocultural situations (e.g., cultural transition and acculturation) [20, 21, 23–25].

More studies have focused on depression in women than depression in men [25]; consequently, male depression has been neglected [18]. Additionally, the overreporting of depression by women and underreporting by men is an artifact of gender-difference research [26, 27]. Moreover, perceived gender differences in depression may be a result of gender stereotypes [28, 29] or methodological issues [9, 18, 27]. These factors may explain confounding effects on gender differences, particularly the higher prevalence of depression in women.

Previous epidemiological studies have analyzed gender and depression in representative Indonesian populations [7, 8]. However, they did not focus on gender and do not provide a clear explanation of gender differences in depression. Therefore, this study investigated gender differences in depression in Indonesia, particularly the possible role of confounding effects.

## 2. Methods

### 2.1. Participants

From June to November 2019, a cross-sectional study was conducted among the general population in Indonesia, which followed the STROBE Statement (https://www.strobe-statement.org/index.php?id=available-checklists) for observational studies. For convenience, the sample comprised individuals living up to 10 km from Hasanuddin University, Makassar, South Sulawesi. This study enrolled 300 male and 300 female volunteers. Participants were given self-report questionnaires after providing written informed consent (response rates for men and women: 88.3% and 81%, respectively). Participants who resigned from participation during the sampling process or did not return their questionnaires were withdrawn from the study. The ethics commission of the Faculty of Medicine at Hasanuddin University approved all procedures (approval number: 616/UN4.6.4.5.31/PP36/2019).

### 2.2. Assessments

The questionnaires comprised items on demographic characteristics, the Cultural Orientation Scale (COS) [22], and the Center for Epidemiological Studies Depression Scale (CES-D) [30]. Both the COS and the CES-D are appropriate measures of dimensions (horizontal–vertical) of individualism–collectivism cultural orientation and perceived depression symptoms, respectively [8, 31–33]. The COS dimensions are horizontal individualism (HI; *my personal identity, independent of others, is very important to me*; *α* = 0.849), vertical individualism (VI; *competition is the law of nature*; *α* = 0.761), horizontal collectivism (HC; *I feel good when I cooperate with others*; *α* = 0.738), and vertical collectivism (VC; *parents and children must stay together as much as possible*; *α* = 0.863). The 20-item CES-D (*α* = 0.812) has four factors [30, 34]: depressed affect (e.g., *I felt depressed*), positive affect (e.g., *I felt that I was just as good as other people*), somatic and retarded activity (e.g., *my sleep was restless*), and interpersonal (e.g., *people were unfriendly*). The reliability of the scales was analyzed using Cronbach's alpha.

### 2.3. Analysis Procedures

Proportions and mean differences were analyzed using the nonparametric chi-square (*χ*^2^) and Mann–Whitney *U* tests. A generalized linear model was performed for multivariate analysis of gender differences in depression, adjusting for cultural orientation. Furthermore, the possible effects of stereotypical symptoms were identified by adjusting their variance by either controlling or not controlling for stereotypical symptom variances in the analysis. When not controlling for stereotypical symptom variances, other symptom variances were controlled for. All analyses controlled for the potential confounding effects of demographic characteristics, and “women” was the reference category. Statistical tests were two-sided with a 95% confidence interval (CI; *α* = 0.05). IBM SPSS for Windows, Version 26.0 was used for analysis (IBM Corp., Armonk, NY, USA).

## 3. Results

After withdrawals (35 men, 57 women), 265 men and 243 women remained. There were significant gender differences in most demographic characteristics (all *p* < 0.001) except ethnicity (*p* = 0.714); in cultural orientation, HI (*p* = 0.003) and VC (*p* < 0.001) evidenced significant gender differences (Table 1).

Table 2 shows no gender difference in overall depression scores (*p* = 0.273) and in scores on the four depression factors (depressed affect, *p* = 0.196; positive affect, *p* = 0.805; somatic and retarded activity, *p* = 0.761; interpersonal, *p* = 0.370). The only gender differences were found in the symptoms, “crying” (*p* = 0.011), “sad” (*p* = 0.015), “cannot get going” (*p* = 0.018), and “people were unfriendly” (*p* = 0.030).

Table 3 shows the multivariate analysis of gender differences in depression. After controlling for potential demographic confounding, no gender differences were found, after adjusting for cultural orientation in the overall and four-factor depression symptoms. However, the symptoms “crying,” “cannot get going,” and “people were unfriendly” remained significant (adjusted odds ratio (ORadj) = 0.597; 95%CI = 0.404–0.828, *p* < 0.01, ORadj = 0.411; 95%CI = 0.230–0.733, *p* < 0.05, ORadj = 0.748; 95%CI = 0.581–0.963, *p* < 0.05, respectively). Men had a higher OR than women for overall scores (ORadj = 1.077; 95%CI = 1.031–1.126, *p* < 0.01) and depressed affect (ORadj = 1.186; 95%CI = 1.043–1.350, *p* < 0.01), particularly “loneliness” (ORadj = 1.464; 95%CI = 1.083–1.978, *p* < 0.05), after adjusting for variance on the three symptoms. Conversely, men had a lower OR than women for overall scores (ORadj = 0.643; 95%CI = 0.527–0.783, *p* < 0.001), depressed affect (ORadj = 0.545; 95%CI = 0.386–0.769, *p* < 0.01), somatic and retarded activity (ORadj = 0.528; 95%CI = 0.304–0.918, *p* < 0.05), and interpersonal (ORadj = 0.745; 95%CI = 0.579–0.960, *p* < 0.05), when only the 17-item variance was adjusted.

## 4. Discussion

### 4.1. Methodological Confounding Effects

No gender differences were found for overall depression and for most of the four-factor depression symptoms. This finding contradicts a previous finding of gender differences in overall and four-factor depression symptoms [35]. Hawkins et al. [36] partly support the finding in the present study of no gender differences except for depressed affect.

These contradictions may indicate methodological confounding factors (e.g., sample preferences) [9, 18], which may affect gender differences. More specifically, both previous studies enrolled samples more vulnerable to depression (married people and students) than the sample (general population) in the present study [11, 37, 38]. In support of this view, some general population studies show no gender differences in depression [6–8].

### 4.2. Cultural Confounding Effects

No significant gender differences were found in the odds of depression after adjusting for cultural orientation and demographic confounding factors, with one exception, which is discussed below. However, significant gender differences were found in cultural orientation (men were higher on HC and lower on VC and HI than women), which may indicate confounding effects on gender differences in depression. It is assumed that lower scores on collectivism dimensions related to gender may indicate a transition process to corresponding individualism dimensions (one-way transition). Men with relatively low VC may experience vertical transitions to VI, whereas women with relatively low HC may experience horizontal transitions to HI. Additionally, the significant gender differences for HI but not for VI may indicate that the transition process is easier on the horizontal than on the vertical dimension [24]. Although women scored slightly higher on VI than men (nonsignificant), the smaller vertical difference (VC–VI) in men could indicate rapid vertical transition.

The current sociocultural transition (collectivism to individualism) in Indonesian society, especially for those living in towns (e.g., Makassar city) [39–42], may support these assumptions. It may predispose gender responsiveness to manage socialization during the transition that fits with gender roles and value motivations [21]. Hence, men are more likely involved in vertical (hierarchy-mastery) and women in horizontal (egalitarian-harmony) dimensions [21, 23, 33, 43]. Additionally, men may require transition to VI to compete and accomplish more hierarchy-mastery rewards, to fulfill their (internalized) expectations, and to obtain self-authorization [21, 33, 43]. For women, horizontal transition to individualism may be required to show that they are independent and strong, or as a behavioral adjustment to social change [21, 44–46].

The transition toward alternative cultural orientations may challenge gender socialization responsiveness in acculturation and cause depression because it clashes with the embedded culture [20, 47, 48]. Men who require self-authorization or express legal nonconformity may be challenged by normative VC socialization, which emphasizes strict conformity to the authority pattern (e.g., living longer with parents/extended family, obedience to family hierarchy, and sacrifice for ingroup). Women who require self-independence may be challenged by normative HC socialization, which emphasizes ingroup harmonization (e.g., benevolence and sociability) [21, 22, 49–51]. Challenges in socializing to their divergent cultural orientations may cause men and women to experience equivalent levels of depression. Therefore, inconsistencies in gender differences in depression may be found during the transition.

However, the higher HC in men and VC in women may indicate gender-based dimension-counterpart coping strategies to negotiate potential socialization difficulties. This may explain the low depression scores found in the present study (under the general threshold for substantial depression) [30, 32]. Men who experience depression with vertical transition may overcome the situation by maintaining harmonization in socialization (e.g., cooperation and tolerance) [52, 53]. Women who experience depression with horizontal transition may overcome the situation by limiting their goals to family preservation and responsibility, especially regarding children [43, 44].

### 4.3. Stereotypical Symptom Confounding Effects

Gender differences in depression were found in a few symptoms (crying, inability to get going, and feeling that people are unfriendly) even after adjustment. The reported higher depression prevalence in women may indicate typical features (feminine stereotypes) of depression symptoms in women, especially crying [18, 25, 35, 54], which seem incongruent with the prototypical male gender role [17, 18]. Additionally, women's motives typically involve emotional relationships (other-oriented), whereas men's motives are typically more self-oriented [9, 55–57]. This may explain why women are more willing to report their interpersonal problems than men. Men are also more stereotypically proactive than women (e.g., they find it easier to “get going”) [58, 59]. Thus, gender differences in depression may occur only in stereotypical symptoms, not in overall symptoms [28, 29], or may reflect adherence to congruent gender-role stereotypes (e.g., men should avoid all things feminine) [17, 18].

Adherence to traditional male roles may prompt men to suppress feminine stereotypical symptoms, which may explain why men in this study were significantly more depressed (particularly lonelier) than women, after adjusting for feminine stereotypical symptoms (i.e., adjusted 3-item stereotypes) [10, 18, 42, 60]. An unwillingness to show weakness (e.g., crying and inability to get going) and an inclination to self-focused attention (indicated by low scores on the interpersonal symptom “feeling people were unfriendly”) may predispose men to experience more self-isolation or social withdrawal when struggling with depression [18, 60]. In contrast, women may express more feminine stereotypical symptoms to obtain support or as social signals (e.g., relationship reconciliation) [53, 60]. Newmann [54] has suggested that this is a gender-typical coping pattern that works better for women.

The odds of depression in women were higher for depressed affect, somatic and retarded activity, and interpersonal problems when feminine stereotypical symptoms were not controlled for (i.e., adjusted only for 17 items). This may indicate that gender differences are not real but artifacts [26, 27] and apparent only for stereotypical symptoms. This stereotypical-induced artifact may lead women to overreport depression and to therefore seem more depressed than men.

### 4.4. Limitations and Future Directions

There were some study limitations. First, it may be premature to assume the existence of a transition process based only on interpretation of a “decline score” in certain cultural dimensions, particularly given the limitations of the study design. Second, the sample is not representative of all residential conditions of the Makassar population, and problems of language comprehension in older participants may have led to biased information. However, these findings may provide preliminary information for further research (with better study designs and more advanced analysis) on possible cultural transition effects on gender differences in depression. Additionally, studies comparing different samples (e.g., general population vs. college students or married couples) would be useful to further investigate confounding effects on gender differences in depression. The possibility of stereotypical symptoms should be considered in gender-difference analysis and diagnosis because these may confound results. Finally, more representative sampling across different residential conditions of the population is needed.

## 5. Conclusions

No gender differences in depression were found, which may indicate methodological issues or the effects of cultural orientation transition. However, for some depression symptoms, there were significant differences in gender, which may indicate the effects of stereotypical symptoms. These may show possible confounding effects on the association between gender and depression. Additionally, the low depression scores for both genders may reflect dimension-counterpart coping strategies that people implement during cultural orientation transitions. Further research is required to build on these preliminary findings.

## Figures and Tables

**Table 1:** Demographic characteristics and cultural orientation.

Variables	Women = 243	Men = 265	*p* value
*Demographic characteristics*			
Age	36.65 ± 11.83	32.63 ± 11.23	<0.001
Ethnicity			
Bugis/Makassar/mixed	86.4	85.3	0.714
Other	13.6	14.7	
Education level			
Low	16.9	4.9	<0.001
Medium	28.8	22.6	
High	54.3	72.5	
Occupation			
Civil servant	7.8	11.7	<0.001
Self-employed	31.7	47.2	
Other^a^	60.5	41.1	
Household income level			
Low	32.5	25.7	<0.001
Medium	44.4	59.2	
High	5.3	10.2	
Unknown	17.7	4.9	
*Cultural orientation*			
Horizontal individualism (HI) (*α* = 0.849)	26.33 ± 7.96	24.92 ± 7.08	0.003
Vertical individualism (VI) (*α* = 0.761)	25.20 ± 7.17	24.82 ± 6.69	0.277
Horizontal collectivism (HC) (*α* = 0.738)	25.71 ± 6.80	26.00 ± 5.49	0.650
Vertical collectivism (VC) (*α* = 0.863)	30.35 ± 6.57	28.69 ± 5.90	<0.001

Data are means ± standard deviations and percentages and were analyzed using chi-square and Mann–Whitney *U* tests (*α* = 0.05). The reliability was analyzed using Cronbach's alpha (*α*). ^a^Occupations with low frequencies were categorized as “other”.

**Table 2:** Mean gender differences in overall and four-factor depression symptoms.

Depression symptoms (CES-D)	Women = 243	Men = 265	*p* value
Overall (0–60)^†^	14.71 ± 8.00	13.98 ± 7.82	0.237
Depressed affect (0–21)	3.76 ± 3.79	3.42 ± 2.82	0.196
Blues (03)^††^	0.55 ± 0.84	0.61 ± 0.87	0.407
Depressed (06)	0.54 ± 0.82	0.58 ± 0.82	0.451
Failure (09)	0.36 ± 0.72	0.34 ± 0.76	0.609
Fearful (10)	0.53 ± 0.75	0.45 ± 0.76	0.100
Lonely (14)	0.55 ± 0.84	0.61 ± 0.84	0.296
Crying (17)	0.54 ± 0.85	0.33 ± 0.66	0.011
Sad (18)	0.69 ± 0.88	0.50 ± 0.76	0.015
Positive affect^a^ (0–12)	3.28 ± 3.02	3.05 ± 2.56	0.805
Good (04)	0.87 ± 1.06	0.79 ± 0.99	0.580
Hopeful (08)	0.81 ± 1.01	0.86 ± 0.94	0.233
Happy (12)	0.89 ± 0.90	0.81 ± 0.84	0.345
Enjoy (16)	0.71 ± 1.01	0.59 ± 0.83	0.544
Somatic and retarded activity (0–21)	6.37 ± 3.47	6.40 ± 2.98	0.761
Bothered (01)	0.54 ± 0.81	0.49 ± 0.70	0.974
Appetite (02)	0.66 ± 0.83	0.66 ± 0.79	0.867
Mind (05)	0.97 ± 0.92	1.04 ± 0.89	0.245
Effort (07)	2.23 ± 0.98	2.28 ± 0.94	0.680
Sleep (11)	0.71 ± 0.91	0.68 ± 0.89	0.852
Talk (13)	0.79 ± 0.86	0.87 ± 0.88	0.330
Get going (20)	0.48 ± 0.50	0.37 ± 0.48	0.018
Interpersonal (0–6)	1.30 ± 1.55	1.10 ± 1.29	0.370
Unfriendly (15)	0.76 ± 1.02	0.54 ± 0.85	0.030
Dislike (19)	0.55 ± 0.83	0.57 ± 0.78	0.484

Mann–Whitney *U* test with *α* = 0.05. Cronbach's alpha (*α*) for the CES-D was 0.812. *Abbreviation*: CES-D: Center for Epidemiological Studies Depression Scale. ^a^Scored in reverse. ^†^Scale range. ^††^This term and all subsequent descriptive terms in the column were item codes in the scale.

**Table 3:** Multivariate analysis of gender differences in overall and four-factor depression symptoms.

Variables	B (SE)	OR (95% CI)
*Overall symptoms*		
Adj. for cultural orientation^a^	−0.002 (0.013)	0.998 (0.973–1.024)
Adj. for cultural orientation + 3‐item stereotypes^b^	0.075 (0.023)	1.077 (1.031–1.126)^∗∗^
Adj. for cultural orientation + 17 items^c^	−0.442 (0.101)	0.643 (0.527–0.783)^∗∗∗^
^a^AIC = 676.147, BIC = 722.683; ^b^AIC = 662.849, BIC = 722.076; ^c^AIC = 663.671, BIC = 782.125		
*Four factors*		
Adj. for cultural orientation		
Depressed affect	0.003 (0.047)	1.004 (0.916–1.099)
Positive affect	−0.008 (0.038)	0.992 (0.921–1.068)
Somatic and retarded activity	0.022 (0.055)	1.021 (0.917–1.139)
Interpersonal	−0.095 (0.081)	0.910 (0.777–1.065)
AIC = 680.490, BIC = 739.717		
Adj. for cultural orientation + 3‐item stereotypes		
Depressed affect	0.171 (0.066)	1.186 (1.043–1.350)^∗∗^
Positive affect	−0.004 (0.039)	0.996 (0.922–1.076)
Somatic and retarded activity	0.118 (0.063)	1.125 (0.994–1.274)
Interpersonal	0.105 (0.143)	1.110 (0.839–1.470)
AIC = 665.942, BIC = 737.861		
Adj. for cultural orientation + 17 items		
Depressed affect	−0.607 (0.176)	0.545 (0.386–0.769)^∗∗^
Positive affect	−0.207 (0.154)	0.813 (0.601–1.100)
Somatic and retarded activity	−0.639 (0.282)	0.528 (0.304–0.918)^∗^
Interpersonal	−0.294 (0.129)	0.745 (0.579–0.960)^∗^
AIC = 663.987, BIC = 790.902		
*Items on the four factors*		
Depressed affect		
Blues	0.200 (0.148)	1.221 (0.913–1.634)
Depressed	0.321 (0.167)	1.379 (0.993–1.915)
Failure	0.189 (0.189)	1.208 (0.833–1.753)
Fearful	−0.251 (0.181)	0.788 (0.546–1.110)
Lonely	0.381 (0.148)	1.464 (1.083–1.978)^∗^
Sad	−0.258 (0.180)	0.773 (0.534–1.118)
Positive affect		
Good	−0.017 (0.122)	0.983 (0.774–1.248)
Hopeful	0.200 (0.126)	1.221 (0.955–1.562)
Happy	−0.096 (0.153)	0.908 (0.674–1.225)
Enjoy	−0.191 (0.154)	0.826 (0.611–1.116)
Somatic and retarded activity		
Bothered	−0.240 (0.153)	0.787 (0.583–1.061)
Appetite	0.204 (0.148)	1.226 (0.916–1.640)
Mind	0.204 (0.133)	1.227 (0.945–1.593)
Effort	0.059 (0.136)	1.061 (0.812–1.386)
Sleep	0.293 (0.153)	1.340 (0.994–1.808)
Talk	0.248 (0.135)	1.281 (0.983–1.670)
Interpersonal		
Dislike	0.224 (0.158)	1.251 (0.918–1.248)
3-item stereotypes		
Crying	−0.547 (0.178)	0.597 (0.404–0.828)^∗∗^
Get going	−0.890 (0.296)	0.411 (0.230–0.733)^∗^
Unfriendly	−0.290 (0.128)	0.748 (0.581–0.963)^∗^
Cultural orientation		
Horizontal individualism (HI)	−0.039 (0.018)	0.962 (0.928–0.996)^∗^
Vertical individualism (VI)	0.026 (0.019)	1.025 (0.985–1.065)
Horizontal collectivism (HC)	0.062 (0.025)	1.063 (1.012–1.116)^∗^
Vertical collectivism (VC)	−0.078 (0.025)	0.926 (0.883–0.971)^∗∗^
AIC = 663.972, BIC = 790.887		

Reference category: women. *Note*: three items that seemed to indicate stereotypical symptoms were grouped as 3-item stereotypes. Adjusting for 3-item stereotypes and 17 items, respectively, means controlling/not controlling for mean stereotypical symptom variances in total depression scores. The scores on the four factors were not analyzed separately. Loneliness symptoms were not significant after excluding 3-item stereotypes from the analysis. HI, HC, and VC were significant in all adjusted models with no substantial change in OR (their respective results are shown at the bottom of the table). All analyses controlled for demographic confounding. The omnibus test was significant for all models (*p* < 0.001). Goodness of fit was measured using the AIC and BIC. *Abbreviation*s: Adj.: adjusted; B: beta coefficient; SE: standard error; OR: odds ratio; CI: confidence interval; AIC: Akaike information criterion; BIC: Bayesian information criterion. ^∗∗∗^*p* < 0.001. ^∗∗^*p* < 0.01. ^∗^*p* < 0.05.

## Data Availability

All data underlying the results are available as part of the article, and no additional source data are required. Additionally, the raw data used to support the findings of this study are available from the corresponding author upon reasonable request.
